# Hybrid CT Angio Suite for Acute Ischemic Stroke: A New Time-Saving Workflow Model?

**DOI:** 10.3390/jcm14030963

**Published:** 2025-02-03

**Authors:** Pietro Trombatore, Simone Cottonaro, Iacopo Valente, Emilio Lozupone, Luigi Della Gatta, Alfio Cannella, Clara Di Lorenzo, Antonio Ragusa, Luca Mammino, Gianluca Galvano

**Affiliations:** 1 Department of Diagnostic Imaging, Interventional Radiology and Neuroradiology, ARNAS Garibaldi, 95123 Catania, Italy; 2 UOSA Interventional Neuroradiology, Fondazione Policlinico Universitario A. Gemelli IRCCS, 00168 Rome, Italy; 3 Department of Neuroradiology, Vito Fazzi Hospital, 73100 Lecce, Italy; 4 UOSD of Neuroradiology, AORN Sant’Anna e San Sebastiano, 81100 Caserta, Italy

**Keywords:** stroke, hybrid suite, mechanical thrombectomy, new technology, time-saving, direct transfer to angio suite

## Abstract

**Objectives:** Explore the effect of the introduction of a hybrid CT angio suite on the in-hospital workflow time of patients with acute ischemic stroke. **Methods:** This was a retrospective observational case-control study. All consecutive patients admitted to our emergency department with suspected ischemic stroke who underwent stroke imaging and mechanical thrombectomy (MT) in the new hybrid CT angio suite from October 2023 to March 2024 were included in the study. The primary outcome was the evaluation of in-hospital workflow times by the assessment of both the time from hospital admission to the beginning of the endovascular treatment (door-to-groin time, DTG) and the time from the interpretation of imaging to arterial puncture (CT-to-groin time, CTTG). The secondary aim was the evaluation of the clinical outcome through the evaluation of the mRS at 3 months. These data were compared to the control group. **Results:** Between October 2023 and March 2024, 50 consecutive patients with suspected ischemic stroke underwent neuroimaging and MT in the hybrid CT angio suite. We observed a significant reduction of the median DTG time from 71 min to 36 min (*p* < 0.001) and the median CT-to-groin time from 44 min to 12 min (*p* < 0.001) compared to the control group. **Conclusions:** The introduction of the hybrid CT angio suite dedicated to acute ischemic stroke has definitely reduced in-hospital delays, allowing better management of these patients.

## 1. Introduction

Stroke is the second leading cause of death worldwide, responsible for 11.6% of global mortality, and ranks as the third leading cause of both death and disability combined [1].

Mechanical thrombectomy (MT) is currently the standard of care for acute ischemic stroke due to large vessel occlusion (LVO) [2,3], as demonstrated by several trials [4,5,6,7,8] and their meta-analysis [9,10].

Several factors may affect the clinical outcome of these patients, but one of the most relevant is the time from onset to reperfusion [10,11].

Pre-hospital time delays represent a substantial portion of the overall treatment timeline. This has historically prompted a focus on pre-hospital pre-notification, rapid neurological assessment, and direct-to-CT (DTCT) transfers, bypassing the emergency department for patients with suspected acute ischemic stroke (AIS). However, despite the effort performed in the last years, pre-hospital delays are notoriously more challenging to influence because they depend not only on the hospital organization but on the entire territorial network [12,13].

Therefore, many strategies have been investigated to reduce the in-hospital workflow time for patients with acute ischemic stroke.

One of the most recent approaches is direct transfer to the angio suite (DTAS): patients with suspected acute ischemic stroke are directly transferred to the angio suite, bypassing CT examination, thanks to new technologies that allow for the acquisition of reasonably acceptable pretreatment imaging of the brain and major intracranial vessels using the flat panel of the angio suite.

**Figure 1:**
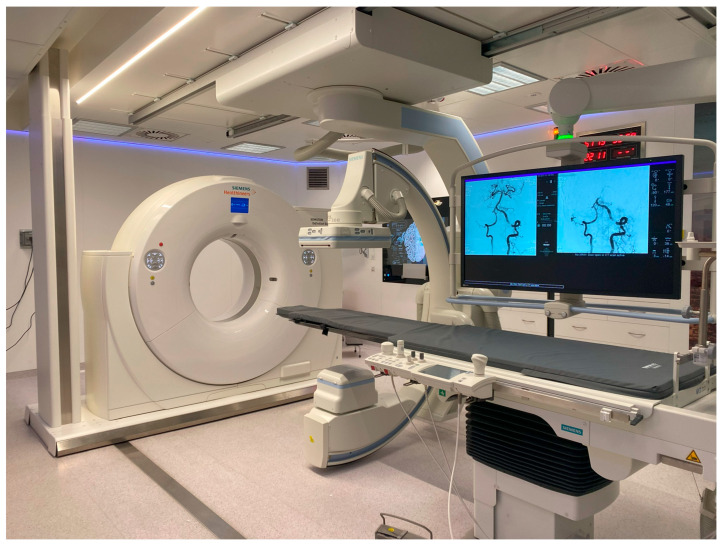
Hybrid CT angio suite (Nexaris Angio-CT, Siemens).

The model we sought to evaluate is comparable to DTAS and is based on a hybrid CT angio suite (Nexaris Angio-CT, Siemens) (Figure 1). This suite combines the advantages of high-resolution CT scanning (Somatom Definition Edge Sliding Gantry CT, 128 slices) with those of a modern angio suite (Artis Zee with Pure) in the same room. All patients with suspected stroke are directly transferred from the emergency room to the dedicated hybrid CT angio suite, where stroke protocol imaging is performed using the CT scan (unenhanced CT, multiphase angio-CT, and CT perfusion). If the criteria for treatment are met, MT, and potentially even thrombolysis, can be directly performed in the same room without the need to move the patient to another location.

This new model is designed to eliminate the time required to transfer the patient from the CT room to the angio suite, thus shortening the time from imaging interpretation to the start of MT (CT-to-groin time, CTTG). As a result, the time from emergency department admission to arterial puncture (door-to-groin time, DTG)—one of the most widely used metrics to evaluate in-hospital workflow efficiency—can also be reduced [14].

The aim of this report is to assess whether this new workflow model can improve the DTG time and clinical outcomes for patients with acute ischemic stroke, comparing it to the conventional neuroimaging in-hospital workflow, in which patients are transferred from the emergency room to the CT room and then to the angio suite.

## 2. Materials and Methods

### 2.1. Study Design

This study was a retrospective observational study.

All consecutive patients admitted to the emergency department of the ARNAS Garibaldi Hospital with suspected acute ischemic stroke with NIHSS ≥ 6 who underwent stroke imaging and MT in the hybrid CT angio suite from October 2023 to March 2024 were included in the study.

As the control group we used the endovascularly treated patients presenting with NIHSS ≥ 6 who were evaluated with a standard CT scan and then transferred to the angio suite from January to June 2023, before the activation of the hybrid CT angio suite.

All patients transferred from another hospital with external neuroimaging were excluded.

All procedures performed were in accordance with the ethical standards of the institutional and/or national research committee and with the 1964 Helsinki Declaration and its later amendments or comparable ethical standards.

Data were analyzed from an Institutional Review Board–approved database.

Clinical data such as relevant workflow times (symptoms onset, hospital admission, imaging acquisition, arterial puncture, and reperfusion), NIHSS on admission, and pre-stroke mRS score were recorded.

Imaging data such as baseline ASPECT score, location of vessel occlusion, CT perfusion data, and reperfusion status using the modified Thrombolysis in Cerebral Infarction score (mTICI) were also recorded.

The primary outcome was the evaluation of in-hospital workflow times by assessment of both the time from hospital admission to the beginning of the endovascular treatment (door-to-groin time, DTG) and the time from the interpretation of imaging to arterial puncture (CT-to-groin time, CTTG); these data were compared with the control group.

Clinical outcome was assessed by the modified Rankin score (mRS) at 3 months from the treatment; mRS score < 3 at 3 months was considered a good clinical outcome.

Statistical analysis was performed using STATA 15.1. Continuous data were reported with medians and interquartile ranges whereas categorical data were expressed as percentages. Continuous variables were compared using the Mann Whitney U-test while for categorical measures, frequencies and percentages were compared with the X 2 test or Fisher exact-test, as appropriate. *p* < 0.05 was considered to be statistically significant.

### 2.2. The New Workflow Model

All patients with suspected ischemic stroke and NIHSS ≥ 6, assessed by a neurologist, are directly transported from the emergency room to the hybrid CT angio suite (Nexaris Angio-CT, Siemens, Munich, Germany).

A neurovascular team, consisting of a neuroradiologist, a specialized nurse, and a radiographer (healthcare professional specialized in the acquisition of X-ray imaging), is available 24/7 in the emergency radiology department to manage stroke cases.

Once the patient arrives in the hybrid CT angio suite, an unenhanced CT scan is first performed to exclude intracranial hemorrhage or large established ischemic lesions that would contraindicate MT. A multiphase angio-CT is then conducted to confirm the presence of large vessel occlusion (LVO). This consists of an initial scan extending from the aortic arch to the vertex, followed by two additional phases focused on the intracranial circulation. Furthermore, CT perfusion is always performed if the time from symptom onset exceeds 6 h. Before 6 h from onset, CT perfusion is conducted only in selected cases, if deemed necessary by the neuroradiologist. Imaging post-processing is performed using a dedicated workstation and software platform (Syngo.via, Siemens).

If indicated, thrombolysis can be initiated immediately after the CT scan in the hybrid CT angio suite.

The indication for MT is determined after imaging evaluation, following updated guidelines [15,16,17]: aged ≥ 18 years; time from onset within 24 h; National Institutes of Health Stroke Scale (NIHSS) score ≥ 6; Alberta Stroke Program Early Computed Tomography Score (ASPECT) score ≥ 6; pre-stroke Modified Rankin Scale (mRS) score ≤ 1.

**Figure 2:**
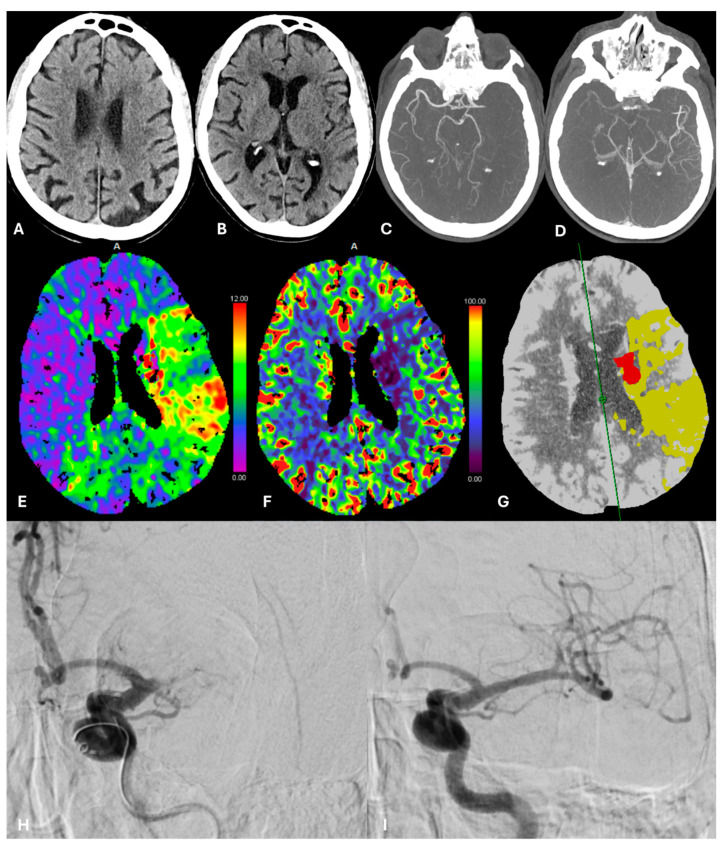
Case Nr. 10. Unenhanced CT shows a small ischemic lesion in the left lentiform nucleus, ASPECT score 9 (**A**,**B**). Multiphase angio-CT documents a left M1 occlusion (**C**) with good collateral circulation in the later acquisition (**D**), also facilitating estimates of the thrombus length. CT Perfusion: Tmax (**E**) and CBF (**F**) maps show a large hypoperfusion area in the left cerebral hemisphere; summary map (**G**) clearly outlines a small ischemic core (red area) and a large ischemic penumbra (yellow area), based on Tmax > 6s and rCBF < 30% respectively. Left M1 occlusion was confirmed during the diagnostic angiography (**H**) and treated by thromboaspiration technique, achieving complete recanalization of the middle cerebral artery (**I**).

If the patient is suitable for MT, the CT gantry is quickly removed, and the angio unit is automatically brought into position, allowing the endovascular team to begin the procedure as soon as possible directly in the hybrid room, without moving the patient (Figure 2).

All procedures were performed under conscious sedation to minimize CTTG time.

## 3. Results

Between October 2023 and March 2024, 50 consecutive patients with suspected ischemic stroke and NIHSS ≥ 6 underwent neuroimaging and MT in the hybrid suite (24M, 26F, median age 76.5 yo, IQR 66–84).

### 3.1. Baseline Characteristic

The median NIHSS was 14 (IQR 7 (10–17)). The median pre-stroke mRS was 0 (range 0–1).

The mean time from symptoms onset to CT was 196 min (3.3 h).

The median ASPECT was 8 (IQR 2 (8–10)). The middle cerebral artery (M1 or M2 segment) was occluded in thirty-four patients, while occlusion of the basilar artery/posterior cerebral artery was detected in seven patients, intracranial internal carotid artery occlusion was found in seven patients, and tandem occlusion was detected in seven patients. Perfusion was performed in 21 patients.

Thrombolysis was performed in 23 patients.

Successful reperfusion (TICI ≥ 2b) was achieved in 44 out of 50 cases (88%).

No procedure-related adverse events were reported.

### 3.2. Outcomes

The median DTG time was 36 min (IQR 8 (32–40)). The median CTTG time was 12 min (IQR 4 (10–14)). These results were compared to the control group, composed of 50 consecutive patients with suspected ischemic stroke and NIHSS ≥ 6 who were evaluated with a standard CT scan and after being transferred to the angio suite for MT from January to June 2023 (19M, 31F, median age 78 yo, IQR 18 (66–84)). We observed a significant reduction of the median DTG time from 71 min to 36 min (*p* < 0.001) and the median CT-to-groin time from 44 min to 12 min (*p* < 0.001) after the introduction of the hybrid CT angio suite (Table 1).

The mRS score at 3 months was recorded. We reported a significant improvement in the clinical outcome with the new workflow model: 29 patients had an mRS < 3 in the case group (58%), compared to 18 patients in the control group (36%) (*p* < 0.05). No differences in terms of baseline ASPECT and recanalization rate (TICI score) were documented between the two groups (Table 1).

## 4. Discussion

The introduction of a dedicated hybrid CT angio suite combined with the new workflow model resulted in a significant improvement in the management of acute ischemic stroke in our hospital, leading to a notable reduction in DTG time and improved clinical outcomes.

Reducing workflow time for patients with acute ischemic stroke has been widely associated with higher rates of successful reperfusion and better clinical outcomes [18]. It is well established that functional independence decreases for every 4-min delay in intrahospital time [11] and for each additional 30-min delay in reperfusion [19,20]. Therefore, guidelines recommend a DTG time of <60 min. [21,22]

However, despite efforts to improve them, door-to-groin times are difficult to reduce below 60–70 min [23,24].

Several schemes have been proposed to reduce the in-hospital workflow time for patients with suspected ischemic stroke, and direct transfer to the angio suite (DTAS) has been widely discussed in recent years. Regardless of the protocol details, several published studies have shown that DTAS significantly decreases DTG time [25,26,27,28]. In particular, Requena et al. recently demonstrated in the ANGIOCAT study that their DTAS protocol was safe and led to decreased in-hospital delays, improving clinical outcomes [29].

Most of the DTAS models reported in the literature involve transferring the patient directly to the angio suite, bypassing the CT scan [26]. These models usually rely on the acquisition of surrogate CT imaging using the flat panel of the angio suite, with all the limitations that this may entail, even with the most modern equipment [25,29,30]. The hybrid CT angio suite appears to overcome these limitations while still offering the time-saving advantages of the traditional DTAS model.

The hybrid CT angio suite allows for high-resolution unenhanced CT imaging, which not only rules out hemorrhagic lesions but also detects recent ischemic lesions, thanks to a precise delineation of grey-white matter differentiation. This facilitates an accurate evaluation of the ASPECT score. In this regard, the ANGIOCAT study recently confirmed that flat panel CT tends to underestimate the ASPECT score compared to conventional neuroimaging, potentially leading to the overtreatment of patients [29].

Another advantage of the hybrid CT angio suite is the ability to perform a complete angio-CT before the procedure, thus avoiding unnecessary cerebral angiography in patients with distal occlusions, lacunar strokes, or stroke mimics, and preventing diagnostic delays in cases of posterior circulation occlusion, where neurological symptoms are often nonspecific and can mimic an anterior circulation LVO. Furthermore, the hybrid suite allows CT perfusion for patients with symptom onsets beyond 6 h and wake-up strokes—an option only feasible with the latest angio suite technology [25,31].

Zhao et al. recently reported a new stroke workflow model (OSSM, one-stop stroke management), based on a hybrid room dedicated to stroke care, similar to ours. The OSSM platform combines computed tomography (CT), magnetic resonance imaging (MRI), and digital subtraction angiography (DSA) equipment in one space, using the same track to transfer patients from one device to another without switching beds. In this study, the author compared the data of patients who received thrombectomy via the OSSM platform with those who underwent the procedure via the traditional workflow. The results of their study demonstrated that the OSSM model significantly reduced in-hospital delays for patients with acute ischemic stroke, thereby improving clinical outcomes [32].

Time savings were also associated with better clinical outcomes in our study: 58% of patients managed by the new workflow model had an mRS score < 3 at 3 months (versus 36% of controls). This improvement is probably related to the reduction in DTG time, but it would not have been achievable without an appropriate in-hospital organization. We believe that for this workflow model to function properly, the hybrid suite must be dedicated to stroke cases. Otherwise, only a small number of cases would benefit from this proposed workflow, especially in high-volume centers. Moreover, the presence of a stroke team available 24 h a day, 7 days a week, is crucial to avoid losing the time savings afforded by the hybrid suite.

Finally, the positive effects of this proposed model on in-hospital times are particularly significant in settings where the angio suite is located far from the CT scanner or in a separate building, as was the case prior to the introduction of the hybrid suite in our hospital.

The economic burden of stroke is significant and the cost associated with this new workflow is one of the most important issues to argue against its application. In addition to the cost of this new technology, in order to efficiently adopt this protocol, hospitals should be able to dedicate the hybrid CT angio suite to the management of stroke patients and to provide a neurovascular team that is always available without interfering with the daily activity. The economic impact of our protocol is probably similar to the DTAS described by Requena et al. that recently demonstrated how their DTAS not only improves clinical outcome, but also decreases costs compared with the standard workflow. The main reason for the cost effectiveness associated with DTAS is the improving of functional outcome, which results in a shorter duration of hospitalization and a reduction in disability-related expenses [33].

Our aim for the future is to perform a cost-effective analysis like the one mentioned above for DTAS to accurately evaluate the economic impact of our workflow in the management of acute ischemic stroke.

Limitations of our study include the observational single-center design, the retrospective nature, the small number of enrolled patients, and the absence of a cost-effectiveness analysis of this new workflow model and of an evaluation of the radiation exposure between the two groups. New prospective randomized long-term studies with a larger number of patients are needed to assess the real impact of a hybrid suite on the reduction of in-hospital workflow time and on the clinical outcome of patients with acute ischemic stroke.

## 5. Conclusions

The introduction of the workflow model based on a hybrid CT angio suite has definitely reduced in-hospital delays, improving the management of the patients with acute ischemic stroke.

## Figures and Tables

**Table 1:** Outcomes. Classic Angio Suite: classic workflow, patient is transferred to the CT scan and then moved to the angio suite. Hybrid Angio Suite: new workflow, patient is directly transferred to the hybrid CT angio suite.

Variables	Classic Angio Suite	Hybrid Angio Suite	*p*-Value
**N**	50	50	
**Age, median (IQR)**	78 (18, 66–84)	76.5 (18, 66–84)	0.81
**baseline NIHSS, median (IQR)**	13.8 (2, 11–13)	14 (7, 10–17)	0.69
**Female**	27 (54%)	26 (52%)	0.84
**Door to CT (min), median (IQR)**	25 (6, 22–28)	23.5 (6, 21–27)	0.19
**CT to Groin Time (min), median (IQR)**	43 (7, 40–47)	12 (4, 10–14)	<0.001
**Door to Groin Time (min), median (IQR)**	69 (10, 63–73)	36 (8, 32–40)	<0.001
**Occlusion Site**			0.62
*Basilar*	7 (14%)	6 (12%)	
*Right M1*	1 (2%)	4 (8%)	
*Left ICA*	2 (4%)	3 (6%)	
*Right M1*	14 (28%)	10 (20%)	
*Left M1*	17 (34%)	13 (26%)	
*Right M2*	2 (4%)	4 (8%)	
*Left M2*	7 (14%)	7 (14%)	
*Left P1*	0 (0%)	1 (2%)	
*Tandem*	0 (0%)	2 (4%)	
**ASPECT baseline, median (IQR)**	8.5 (2, 8–10)	8 (2, 8–10)	0.52
**mTICI 2b/3**	42 (84%)	44 (88%)	0.77
**90-days mRS 0–2**	18 (36%)	29 (58%)	0.03
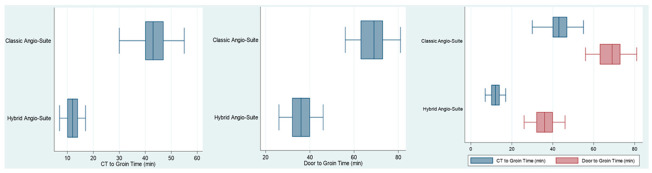			

## Data Availability

The data presented in this study are available on request from the corresponding author.
